# Right Ventricular Perforation Presenting as Tingling of the Left Breast

**DOI:** 10.7759/cureus.7839

**Published:** 2020-04-26

**Authors:** Rony Shah, Andrew Barnes, Fagunkumar Modi, John Royalty, Jeffrey Jordan

**Affiliations:** 1 Internal Medicine, Citrus Memorial Hospital, Inverness, USA; 2 Cardiovascular Surgery, Citrus Memorial Hospital, Inverness, USA

**Keywords:** right ventricular perforation, pacemaker lead displacement, paresthesia of left breast, tingling of left breast

## Abstract

Pacemaker lead-associated cardiac perforation is a rare phenomenon. Lead perforations can be acute, subacute, or chronic following lead placement. Symptoms are typically nonspecific and depend on the location of the displaced lead. Diagnostic workup requires interrogation of the pacemaker and imaging studies. Management of lead displacement is dependent on multiple risk factors such as age, gender, corticosteroid use, and anticoagulation therapy.

A 74-year-old female with a history of myosin light chain kinase (MYLK) 2 hypertrophic cardiomyopathy, Sjogren’s syndrome, Raynaud’s disease, and sick sinus syndrome was evaluated for an abnormal finding on pacemaker interrogation. The patient’s only symptom was tingling of her left breast. Imaging studies confirmed pacemaker lead perforation. Right ventricle perforation due to a pacemaker lead displacement can cause severe complications. Early identification and treatment by physicians can reduce the risk of mortality.

## Introduction

Pacemaker lead-associated cardiac perforation is a rare complication. The incidence of cardiac perforation after pacemaker implantation is 0.1%-0.8% [1]. It is a rare complication that can be fatal if discovered too late. Perforations may be acute (which occur up to 24 hours after implantation, may lead to tamponade or death), subacute, which occur up to one month after implantation, or chronic, which appears after one month [2]. We report a unique case of right ventricular perforation that presented as tingling of the left breast.

## Case presentation

**Figure 1:**
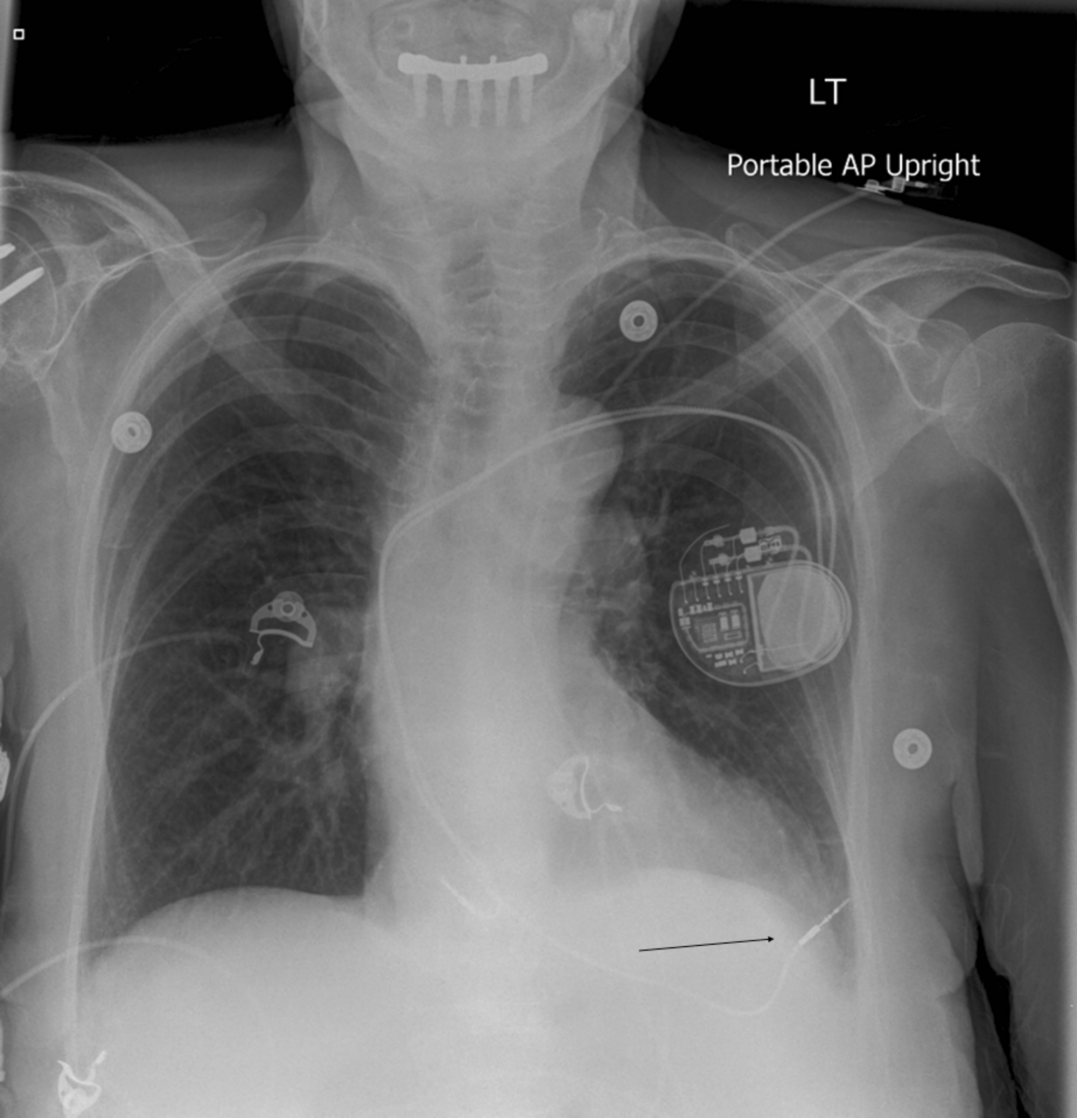
Chest X-ray of displaced lead This image shows a displaced pacemaker lead which has migrated through the myometrium and pericardium into the chest well.

**Figure 2:**
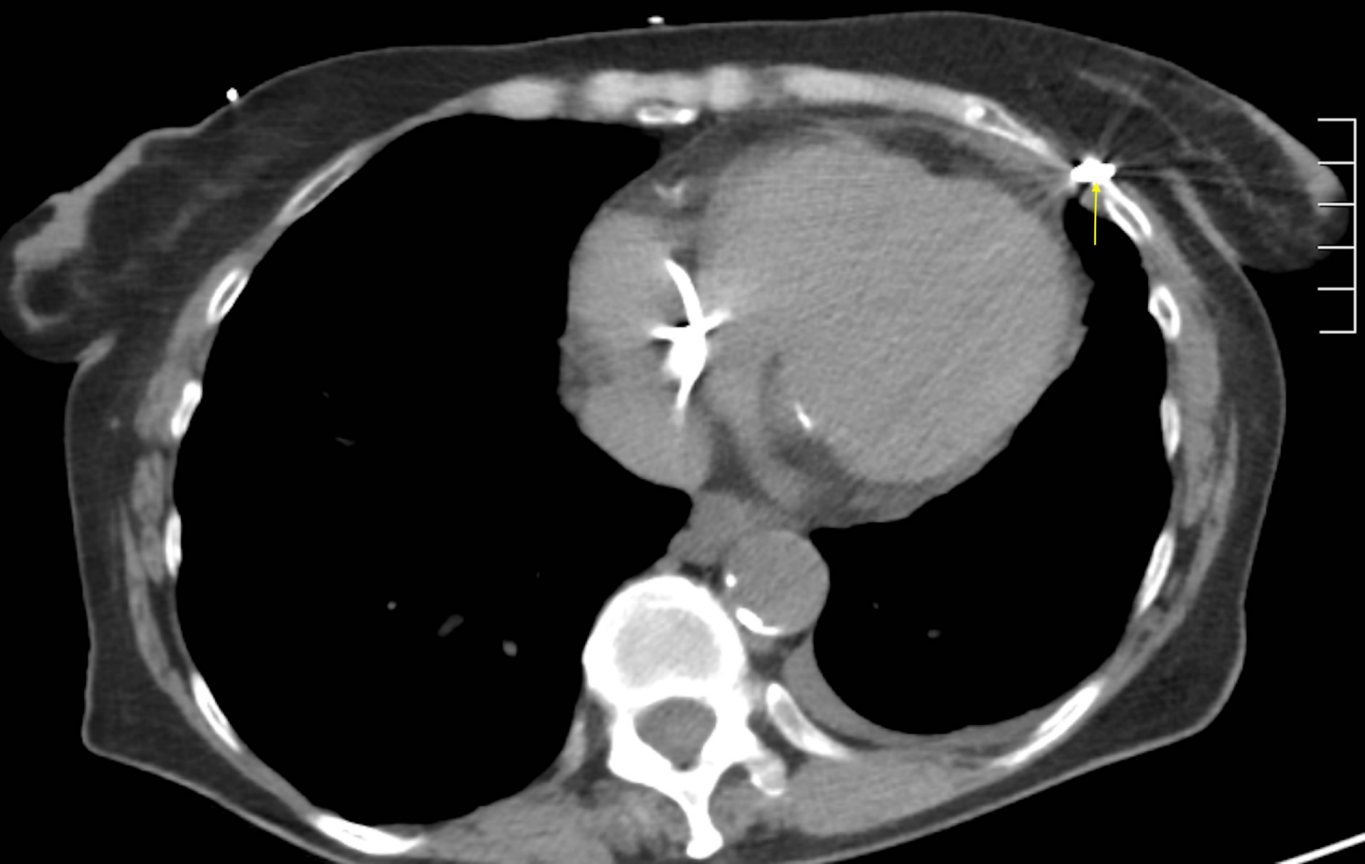
CT chest without contrast This image is a coronal view of a chest CT showing the pacemaker lead penetrating through the intercoastal muscle and into the chest well.

**Figure 3:**
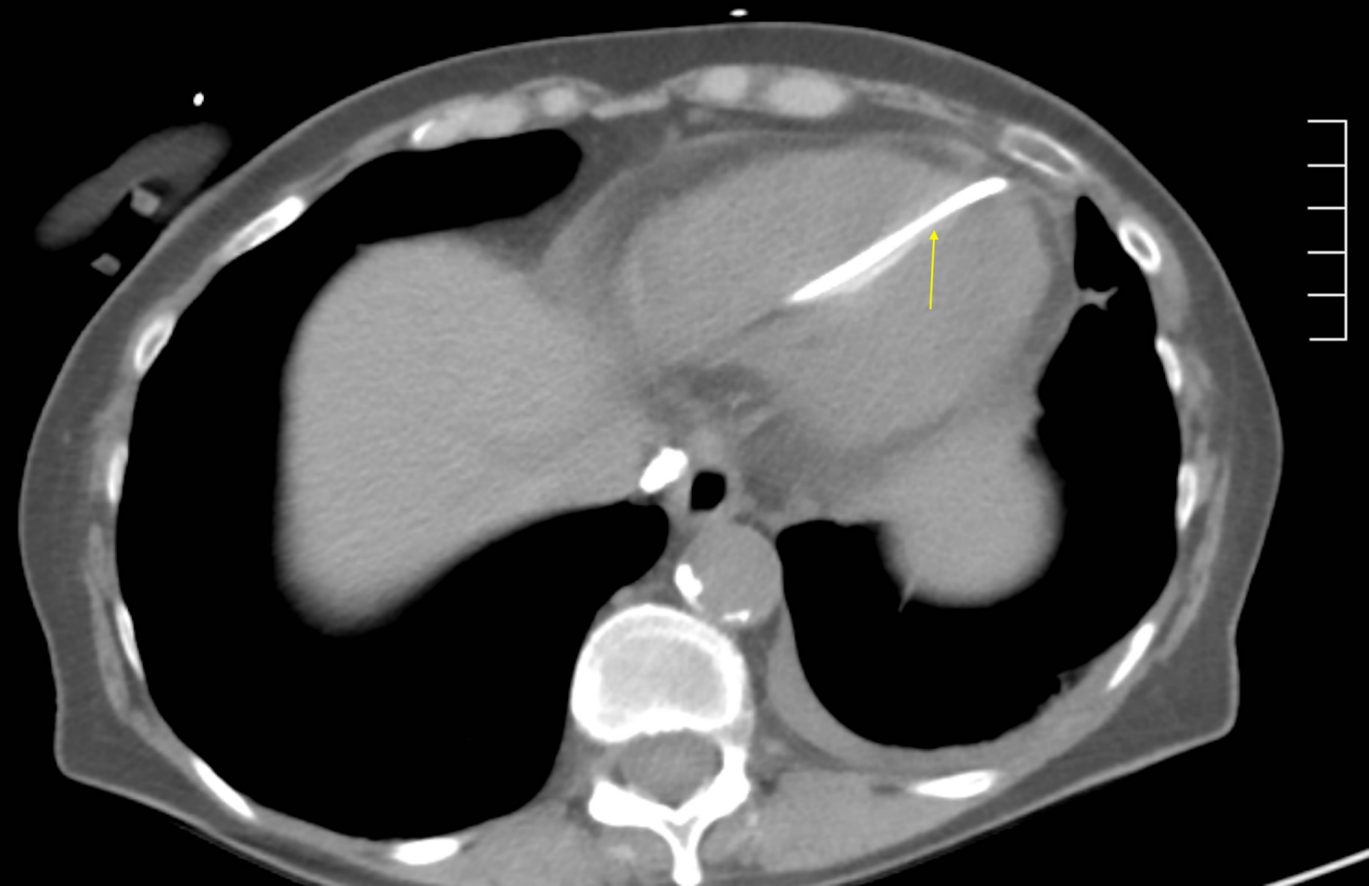
CT chest without contrast This image is a coronal view of a chest CT showing the pacemaker lead penetrating through the right ventricle and pericardium.

**Figure 4:**
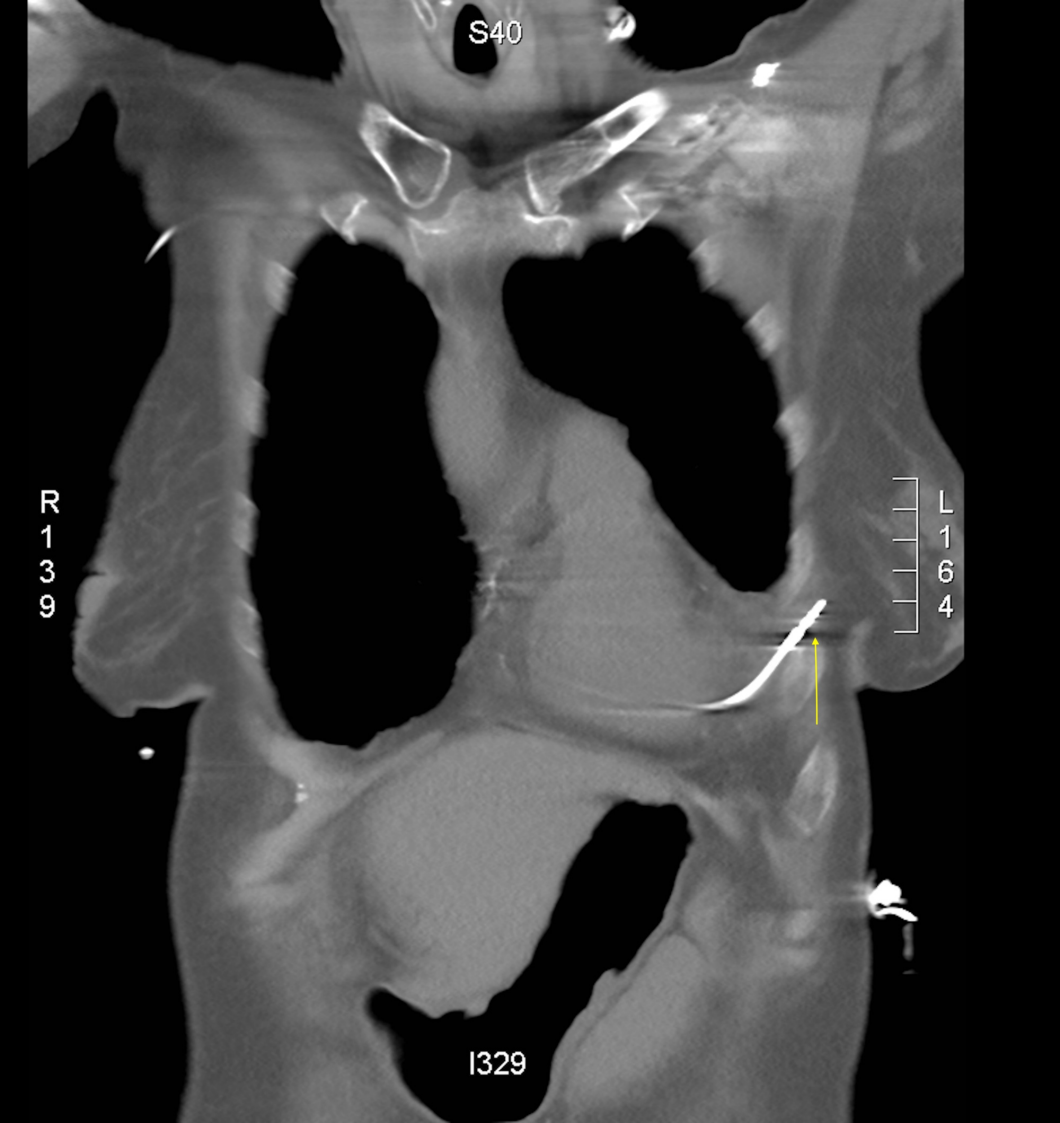
CT chest without contrast This image is a sagittal view of a chest CT showing the pacemaker lead penetrating through the myometrium, pericardium, intercoastal muscle and into the chest well.

**Figure 5:**
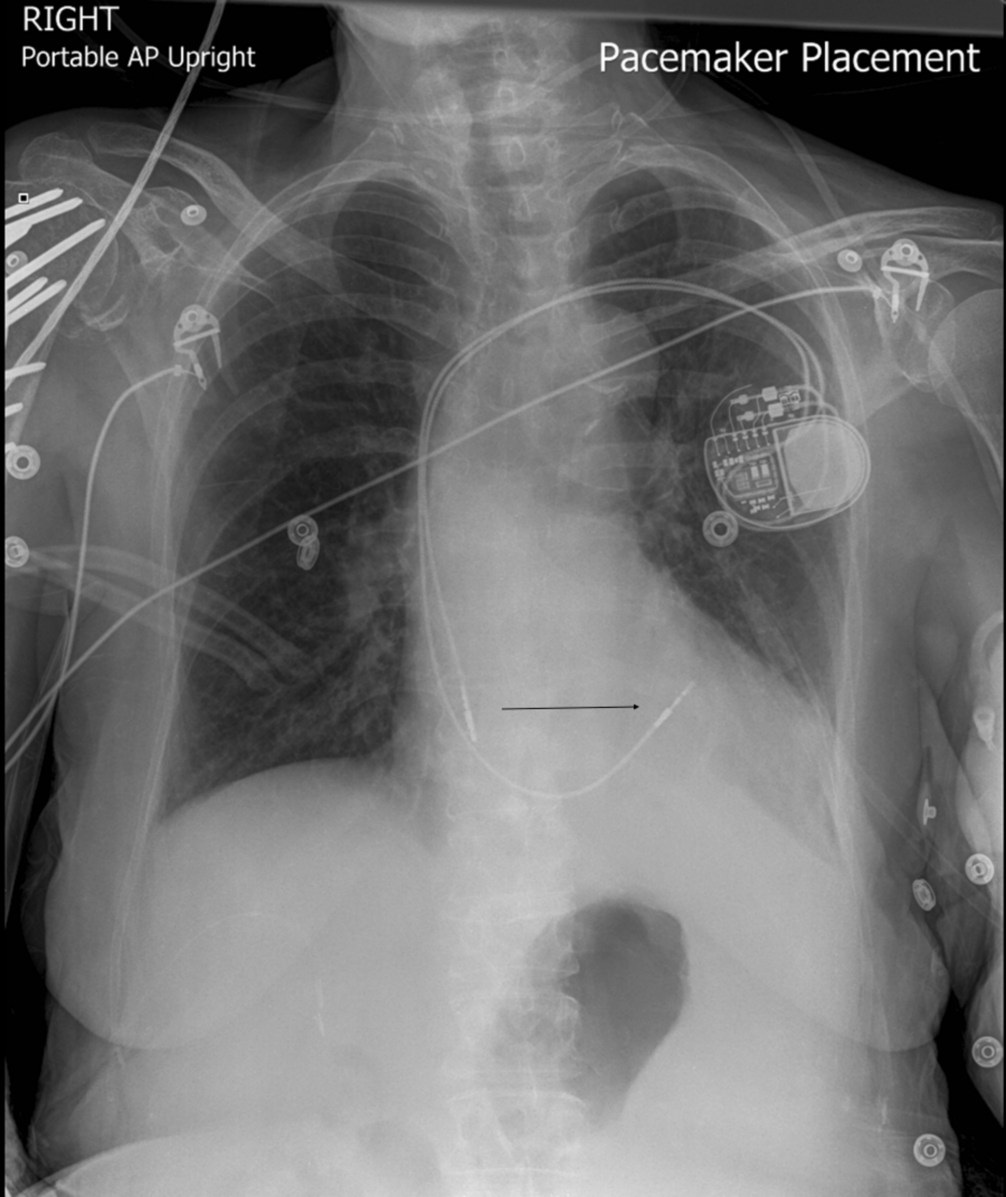
Chest X-ray of repositioned lead This image shows the repositioned pacemaker lead.

A 74-year-old female presented to the emergency room for an abnormal finding of a new pacemaker interrogation. She had a past medical history of myosin light chain kinase (MYLK) 2 hypertrophic cardiomyopathy, Sjogren’s syndrome, Raynaud’s disease, and sick sinus syndrome. The patient had dual-chamber pacemaker placement for sick sinus syndrome. During her two-week pacemaker interrogation, there was less than optimal capture of the ventricular lead. The ventricular lead was sensing atrial P waves suggestive of a possible pullback of the pacemaker lead. The patient's only complaint was a tingling sensation of her left breast. Chest X-ray demonstrated a misplaced right ventricular pacemaker lead (Figure 1). Computed tomography (CT) chest scan without contrast revealed perforation of the right ventricle with a right ventricular lead, lead extension through the myometrium, pericardium, and intercostal muscle into the left chest wall, and a small left pleural effusion (Figures 2-4). The patient underwent repositioning of the right ventricular pacemaker lead with vascular surgery. The ventricular lead was drawn back and passed up into the pulmonary artery and backed down until it settled into the proximal septum. Postoperative monitoring including serial chest X-rays and transthoracic echocardiograms was negative for pericardial effusion, pleural effusion, or pneumothorax (Figure 5). Electrocardiogram (EKG) showed an atrial-ventricular paced rhythm. Repeat EKG the following day showed sinus rhythm with intraventricular conduction delay. The patient was hemodynamically stable and discharged home with a good prognosis.

## Discussion

Right ventricular perforation usually presents during or within 24 hours of implantation and has a prevalence rate of 0.1% to 6% [3]. Symptoms of cardiac perforation can vary, including extracardiac muscle stimulation, chest pain, shortness of breath due to pneumothorax/hemothorax, pericardial tamponade, and pneumomediastinum [3]. The symptoms are nonspecific and depend on the location of the displaced lead in the pericardium, pleural space, lung parenchyma, or chest wall. Our patient’s only symptom was tingling of the left breast due to lead displacement into the left chest wall, which is an unusual symptom of lead perforation. To our knowledge, this is the only case that reported breast tingling as a symptom following lead displacement. Risk factors of lead perforation are temporary pacemaker implantation, corticosteroid use, active fixation leads, low body mass index, old age, female gender, and anticoagulation therapy [3]. The risk factors in our patient were age and gender. Hirschl and colleagues. examined 100 chest CT scans in asymptomatic patients with implantable cardiac devices and found perforations in 15% of cases, more commonly with atrial (15%) than ventricular (6%) leads [4,5]. Perforations due to right ventricular leads occurred significantly more often with defibrillators (14%) than with pacemakers (3%) [4]. Interrogation of the pacemaker device may find abnormalities, which have developed compared to the previous check, suggestive of lead displacement [4]. In our patient, the pacemaker interrogation revealed less than optimal ventricular capture and sensing atrial P wave indicative of possible lead displacement. Suspect perforation on chest X-ray if a lead is abnormally positioned, generally more caudal than usual for ventricular leads [4,6]. The lead displacement can be identified by CT, which is ideally carried out with cardiac synchronization and images reconstructed during diastole [4,6]. Approach to lead displacement is different depending on the time of implantation, clinical status, pacemaker dependency, lead displaced (atrial vs ventricular), and degree of malfunction of the device [7]. In early displacement, reopening the pouch and lead reposition is possible [7]. In late displacement, surgical repositioning is not possible. In late displacement, implementing a new lead in the chamber will cancel the previous one or repositioning via percutaneous access is a less aggressive option [7]. Fortunately, for our patient, repositioning of the lead was uneventful, as no pericardial effusion was noted before or immediately after the procedure.

## Conclusions

Right ventricular perforation secondary to pacemaker lead displacement is rare but it can lead to severe complications. Patients symptoms will vary from case to case due to different complications associated with it. Early identification and treatment can reduce the risk of mortality. The management of pacemaker lead displacement will depend on multiple variables. 
